# Silencing of *GhORP_A02* enhances drought tolerance in *Gossypium hirsutum*

**DOI:** 10.1186/s12864-022-09099-y

**Published:** 2023-01-09

**Authors:** Sani Muhammad Tajo, Zhaoe Pan, Yinhua Jia, Shoupu He, Baojun Chen, Salisu Bello Sadau, Yusuf KM, Aboleri Adijat Ajadi, Mian Faisal Nazir, Umar Auta, Xiaoli Geng, Xiongming Du

**Affiliations:** 1 State Key Laboratory of Cotton Biology, Institute of Cotton Research, Chinese Academy of Agricultural Science, Anyang, China; 2 Bioresources Development Centre, National Biotechnology Development Agency, Abuja, Nigeria; 3 Biotechnology Unit, National Cereal Research Institute, Bida, Nigeria

**Keywords:** *Gossypium hirsutum*, Drought, *ORP* genes, VIGS

## Abstract

**Background:**

*ORP* (Oxysterol-binding protein-related proteins) genes play a role in lipid metabolism, vesicular transferring and signaling, and non-vesicular sterol transport. However, no systematic identification and analysis of *ORP* genes have been reported in cotton.

**Result:**

In this study, we identified 14, 14, 7, and 7 *ORP* genes in *G. hirsutum*, *G. barbadense*, *G. arboreum*, and *G. raimondii*, respectively. Phylogenetic analysis showed that all *ORP* genes could be classified into four groups. Gene structure and conserved motif analysis suggest that the function of this gene family was conserved. The Ka/Ks analysis showed that this gene family was exposed to purifying selection during evolution. Transcriptome data showed that four *ORP* genes, especially *GhORP_A02*, were induced by abiotic stress treatment. The cis-acting elements in the *ORP* promoters were responsive to phytohormones and various abiotic stresses. The silenced plants of *GhORP_A02* were more sensitive to drought stress when compared to control.

**Conclusion:**

The major finding of this study shed light on the potential role of *ORP* genes in abiotic stress and provided a fundamental resource for further analysis in cotton.

**Supplementary Information:**

The online version contains supplementary material available at 10.1186/s12864-022-09099-y.

## Introduction

Cotton is the most important natural fiber crop and amounts to 35% of all fibers produced worldwide [49]. Cotton is a part of the Malvaceae family and belongs to the *Gossypium* genus with 45 diploids and five allotetraploid species found in Africa, America, Galapagos, India, Australia, Arabia, and Hawaii [16]. Eight diploid genomes (A-G and K) are assigned to these 50 species [1, 5]. Abiotic stresses significantly limit cotton growth, output, and development, resulting in a 50% decline in worldwide yield [7, 15]. Abiotic stresses such as drought, salinity, cold, and heat have negative effects on plant photosynthesis and respiration, attributed to disruption of various molecular pathways, such as Ca^2+^ signaling, abscisic acid (ABA) signaling, reactive oxygen species (ROS) metabolism, and sugar and lipid metabolism [6, 10, 18, 27]. Drought is considered as an important yield limiting factor [24, 40]. Cotton plants have evolved a range of sophisticated signaling networks, including metabolic, physiological, and morphological changes, to adapt to drought stress [24]. Drought tolerance mechanisms in cotton include drought avoidance, drought tolerance, drought recovery, and drought escape [14]. These tolerance mechanisms are aided by signal transduction and hormone regulation, such as jasmonic acid (JA), ABA, and ethylene synthesis [11, 13, 14, 20, 26, 32, 34, 52, 56].

*ORP* (oxysterol-binding protein-related proteins) genes play a key role in lipid metabolism, vesicular transferring and signaling, and non-vesicular sterol transport [41]. Previous studies about *ORP* genes in *Arabidopsis*, soybean, and petunia have also demonstrated its significant role in biotic stress, abiotic stress [31, 43, 45]. The *Arabidopsis* genome encodes 12 *ORP* genes, and the rice genome encodes six *ORP* genes [48]. Although the *ORP* gene in plants has been cloned, there are few studies focused on their functions. In *Arabidopsis*, *ORP3a*, located the endoplasmic reticulum, interacts with VAP33 family member PVA12 [43]. In *Petunia inflata*, *PiORP1* participates in pollen growth and development by interacting with PRK1 receptor kinase on the plasma membrane of a hybrid pollen tube [45]. In soybean, the expression of *GmOSBP* was inhibited by salt stress but induced in aging leaves, indicating that *GmOSBP* may be involved in stress response and the cell aging process [31]. In this study, we performed genome-wide identification and investigated phylogenetic relationships, gene structure, conserved domains, gene duplication events and expression files of *ORP* genes. Our study may be useful for the future molecular and biological function of the *ORP* gene family in cotton.

## Materials and methods

### *ORP* gene identification in cotton species

The conserved domain PF15413 of *ORP* genes was obtained from PFAM (http://pfam.xfam.org) and used as a query sequence to retrieve the *ORP* genes in four cotton species by Hmmer 3.0 (http://hmmer.org), and the identity of the *ORPs* genes was analyzed by SMART (http://smart.embl-heidelberg.de/smart/). The physical and chemical characteristics of ORP proteins, including molecular weight, protein length, molecular charge, isoelectric point, and grand average of hydropathy, were obtained from CottonFGD (https://cottonfgd.net/).

### Chromosomal mapping

We used the GFF3 files of the *ORPs* genes downloaded from CottonFGD to find the distribution on all chromosomes. TBtools software (version 1.098685) was then used to visualize the gene’s location on chromosomes [8].

### Phylogenetic tree and collinearity analysis

The full-length protein sequences of ORP genes from *Gossypium* were downloaded and aligned using ClustalW with default settings. The phylogenetic tree was constructed using the neighbor-joining method in MEGA 6 with default parameters and 1000-bootstrap replicates (http://www.megasoftware.net/). The protein sequences of *Gossypium hirsutum* (*G. hirsutum*) have been searched along the protein databases of *Gossypium arboreum* (*G. arboreum*), *Gossypium barbasense* (*G. barbasense*) and *Gossypium raimondii* (*G. raimondii*) by BlastP to identify homologous genes and hits with E-values of 1.0E^–5^ and similarity of 90% were considered noteworthy. TBtools program was used to create the collinearity analysis using the GFF3 file, linked file, and gene IDs. Collinearity analysis was performed among three cotton species (*G. hirsutum, G. arboreum*, and *G. raimondii*) using Circle gene viewer in TBtools software to determine collinear gene pairs. Coding and protein sequences of all homolog genes were used to calculate the Ka/Ks (Non-synonymous substitution- rate/Synonymous substitution rate) value by TBtools [47].

### Gene structure, conserved motif analysis and prediction of regulatory elements

The gene structures were analyzed using a gene structure displayer server (http://gsds.cbi.pku.edu.cn/). Conserved motifs of *ORP* genes were discovered with default settings of the MEME Suite (http://memesuite.org/index.html) [4]. The gene structure was visualized using TBtools (v1.098661) [8]. 1500 bp upstream sequences of *ORPs* genes from different cotton species were downloaded from CottonFGD and uploaded into PlantCare (http://bioinformatics.psb.ugent.be/webtools/plantcare/html/) to identify *cis*-regulatory elements [30].

### Expression profile analysis of *ORP* gene family

FPKM values (fragments per kilobase of exon per million fragments mapped) of *ORP* genes were downloaded from CottonFGD. We analyzed the expression profiles of *ORP* genes under different stress treatments, which included PEG, salt, heat and cold treatments.

### Virus-induced gene silencing (VIGS)

For virus-induced gene silencing, the cotton variety H117 was employed. H177 was developed by the Institute of Cotton Research Anyang of the Chinese Academy of Agricultural Sciences. This Variety was chosen because it is particularly susceptible to many environmental stresses, including drought. A 306-bp fragment of *GH_A02G0809* was amplified from *G. hirsutum* acc. TM-1 with gene-specific primers. The PCR product was then digested with *Spe* I and *Acs* I and cloned into *Spe I-Acs I -Cut pCLCrVA*. The fusion vector was named *pCLCrVA: GhORP_A02* and transformed into *Agrobacterium tumefaciens* strain LBA4404. The control vector *pCLCrVA*, *pCLCrVA: GhORP_A02* and positive vector *pCLCrVA: PDS* were mixed with *pCLCrVB* at a 1:1 ratio [19]. The mixed *Agrobacterium tumefaciens* solutions were injected into the ten-day-old cotton cotyledons on the abaxial side with a needle-free syringe. The plants were placed at room temperature in the dark overnight and grew at 23 °C with a 16 h / 8 h light/dark cycle. *Agrobacterium* infection was carried out three times with 30 plants for each vector. The primers for VIGS vector construction are listed in Table S1. Wild type and the plants injected with *pCLCrVA* empty control and *pCLCrVA: GhORP_A02* were subjected to drought treatment after four weeks. Drought treatments of the seedlings were irrigated with 15% PEG6000, while control plants were irrigated with 1/2 MS nutrient solution.

### RNA extraction and quantitative real-time PCR (qRT-PCR) analysis

Total RNA was extracted from fresh leaves and roots using TRIzol® Plus RNA Purification Kit (Invitrogen, CA) based on the manufacturer’s instructions. Approximately 1 µg RNA was reversely synthesized into cDNA using the iScriptTM Synthesis Kit (Quanta BioSciences, MD). The qRT-PCR was carried out in an Eppendorf real-time PCR equipment using a 5 µl cDNA template (diluted 1/100), 5 µl primers (2.4 M), and 10 µl SYBR green mixture (Promega, Madison, WI). Histone 3 was used as the internal control, and the relative expression levels of the *ORP* gene were calculated by the 2^−ΔΔCt^ method [35].

### Physiological analysis

Physiological parameters, including ion leakage, chlorophyll content, excised leaf water loss, and relative leaf water content, were determined after 10 days of drought treatment. Wild type, silenced, and control plants (ten plants for each) were harvested after drought stress for oxidant and antioxidant concentration analysis. The H_2_O_2_ content, peroxidase (POD), malondialdehyde (MDA) and catalase (CAT) were determined by using the corresponding ROS content reagent kits and enzyme activity kit (Solarbio, China) according to the manufacturer’s instructions. The experiment was repeated three times.

## Results

### Genome-wide identification and chromosomal locations of the cotton *ORP* genes

**Fig. 1:**
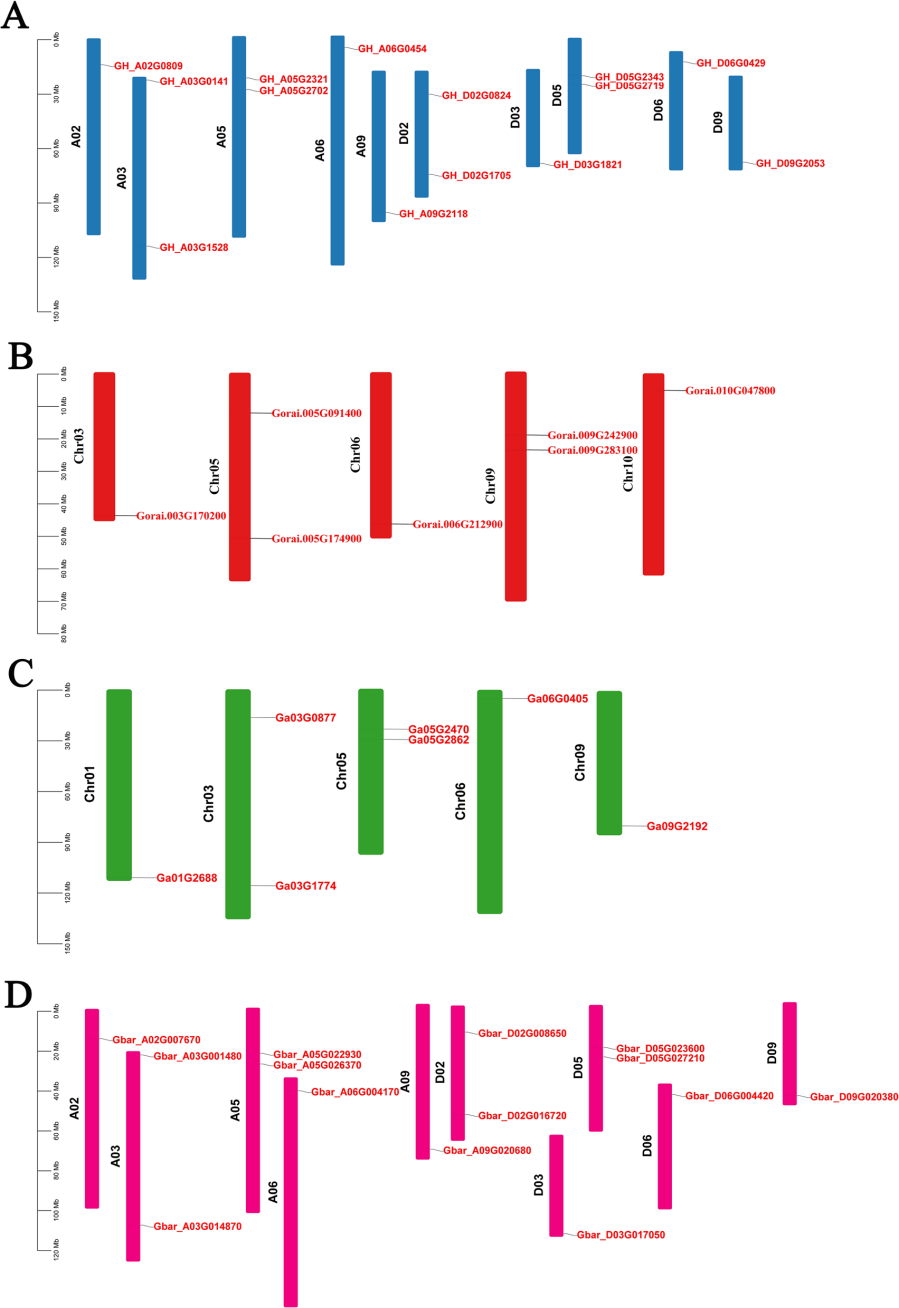
Chromosomal positions of *ORP* genes in *Gossypium *species. **A** *G. hirsutum*, **B** *G. raimondii*, **C** *G. arboreum*, **D** *G. barbadense*. Based on their genome, each species’ chromosomal location was plotted

To identify all *ORP* genes in two allotetraploid cotton, *G. hirsutum* (AD_1_), *G. barbadense* (AD_2_) and its two diploid ancestors *G. arboreum* (AA) and *G. raimondii* (DD), we used conserved domain Pfam 15,413 to retrieve *ORP* genes and identified 42 *ORP* genes. *G. hirsutum*, *G. barbadense*, *G. arboreum*, and *G. raimondii* have 14, 14, seven, and seven *ORP* genes, respectively. The *ORP* gene ID and predicted protein properties and subcellular locations are listed in Table 1. Variable distribution of *ORP* genes on chromosomes across all four cotton species was observed (Fig. 1). In *G. hirsutum* and *G. barbadense*, *ORP* genes were uniformly distributed on the At and Dt chromosome. In *G. hirsutum* and *G. barbadense*, 14 *ORP* genes were located on chromosomes A02, A03, A05, A06, A09, D02, D03, D05, D06 and D09. Two ORP genes were located on chromosomes A03, A05, D02 and D05. In *G. arboreum*, seven *ORP* genes were located on chromosomes A01, A03, A05, A06 and A09. In *G. raimondii*, seven *ORP* genes were located on chromosomes D03, D05, D06, D09 and D10.

**Table 1:** Protein physicochemical properties of *ORP* genes in *Gossypium* species

Gene ID	Protein Length	MW (kDa)	Charge	pI	Subcellular Location
GH_A02G0809	242	27.89	7.0	9.53	nucleus
GH_A03G0141	678	76.64	5.5	7.01	nucleus
GH_A03G1528	802	91.56	-5.50	5.95	nucleus
GH_A05G2321	784	89.83	11.0	8.07	nucleus, chloroplast
GH_A05G2702	826	94.26	13.0	8.04	nucleus
GH_A06G0454	817	93.50	10.5	7.60	nucleus
GH_A09G2118	783	89.67	6.0	7.03	nucleus
GH_D02G0824	238	27.54	8.0	9.76	nucleus, cytoplasm
GH_D02G1705	802	91.48	-5.0	5.98	nucleus
GH_D03G1821	833	95.31	11.0	7.44	chloroplast
GH_D05G2343	810	92.74	14.5	8.34	nucleus, chloroplast
GH_D05G2719	806	91.55	18.0	8.55	nucleus
GH_D06G0429	817	93.47	10.0	7.59	nucleus
GH_D09G2053	764	87.44	3.0	6.76	nucleus
Gbar_A02G007670	241	27.69	7.0	9.58	nucleus, cytoplasm
Gbar_A03G001480	679	76.82	5.5	7.01	nucleus, cytoplasm
Gbar_A03G014870	802	91.57	-3.5	6.16	nucleus
Gbar_A05G022930	741	84.56	9.5	7.84	nucleus, chloroplast
Gbar_A05G026370	765	86.38	11.5	7.93	nucleus
Gbar_A06G004170	817	93.50	10.5	7.60	nucleus
Gbar_A09G020680	783	89.72	6.0	7.04	nucleus
Gbar_D02G008650	259	29.95	9.0	9.42	nucleus, cytoplasm
Gbar_D02G016720	812	92.58	-5.0	5.98	nucleus
Gbar_D03G017050	679	76.80	6.0	7.04	nucleus
Gbar_D05G023600	783	89.69	13.5	8.29	nucleus, chloroplast
Gbar_D05G027210	828	94.16	14.5	8.17	nucleus
Gbar_D06G004420	817	93.34	10.0	7.58	nucleus
Gbar_D09G020380	764	87.41	2.0	6.67	nucleus
Ga01G2688	826	94.49	12.0	7.64	nucleus
Ga03G0877	242	27.91	7.0	9.53	nucleus
Ga03G1774	802	91.63	-5.5	5.95	nucleus
Ga05G2470	828	94.56	15.5	8.38	chloroplast
Ga05G2862	827	94.42	14.0	8.16	nucleus
Ga06G0405	817	93.49	10.0	7.59	nucleus
Ga09G2192	765	87.48	3.0	6.76	nucleus
Gorai.003G170200	767	87.40	2.5	6.71	nucleus
Gorai.005G091400	274	31.87	12.5	9.76	nucleus, cytoplasm
Gorai.005G174900	812	92.62	-4.0	6.09	nucleus
Gorai.006G212900	765	87.51	1.5	6.63	nucleus
Gorai.009G242900	784	89.81	12.5	8.19	nucleus, chloroplast
Gorai.009G283100	827	94.12	12.0	7.90	nucleus
Gorai.010G047800	817	93.46	11.0	7.75	nucleus

### Phylogenetic and collinearity analysis of cotton *ORP* genes

**Fig. 2:**
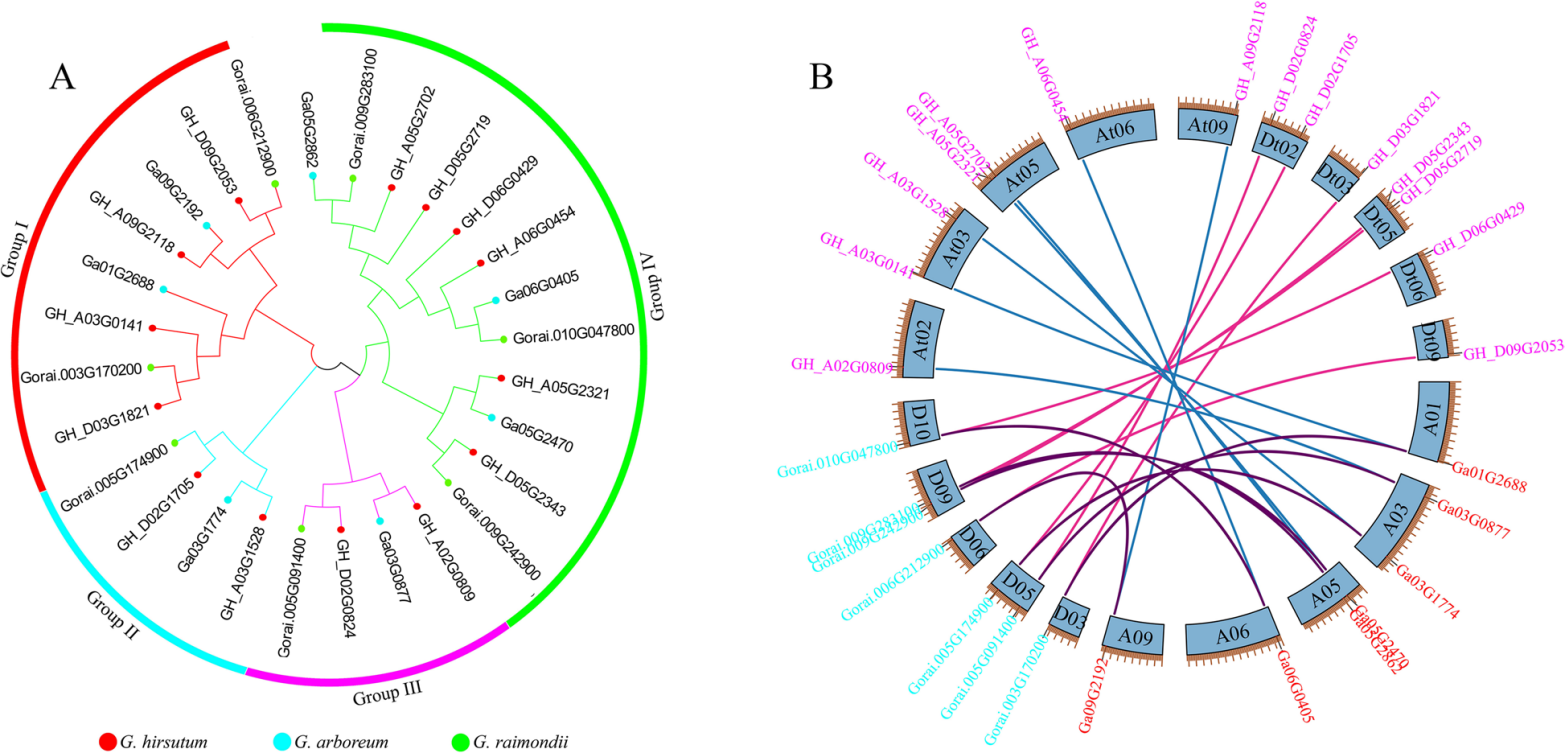
Phylogenetic and collinearity analysis of *ORP* genes. **A** Phylogenetic tree of ORP genes in *G. hirsutum*, *G. arboreum*, *G. raimondii*. **B** The synteny relationships of *ORP* genes among three cotton species. *ORP* genes in *G. hirsutum*, *G. arboreum*, and *G. raimondii* are indicated in pink, red and blue, respectively

According to the phylogenetic analyses, all the *ORP* genes could be classified into four clades (Fig. 2A). Four, two, two, and six *ORP* genes from *G. hirsutum* were classified into Group I to Group IV. Both in *G. arboreum* and *G. raimondii*, Group I, II, III and IV have two, one, one, and three *ORP* genes. To analyze the evolution of the *ORP* genes from diploid to tetraploid species, collinearity analysis was performed among three cotton species (*G. hirsutum*, *G. arboreum*, and *G. raimondii*). There were seven, seven, and seven orthologous gene pairs between the A and D genomes, the At subgenome and the A genome, and the Dt subgenome and the D genome (Fig. 2B). The number and relatedness of *ORP* genes in the three species suggested that *ORP* genes were not lost during *G. hirsutum* speciation.

### Evolution of *ORP* genes in *Gossypium* species

Natural selection has no effect on gene’s *Ka/Ks* values during the evolutionary trend, but *Ka/Ks* > 1, *Ks/Ka* = 1, or *Ka/Ks* < 1, the *Ka/Ks* value indicates positive, neutral, or negative selection, respectively [55]. Similar results were found in the distributions of *Ka*, *Ks*, and *Ka/Ks* among homologous pairs of *Gossypium* species. The *Ka/Ks* ratio of most orthologous gene pairs was less than one, indicating purifying selection during evolution resulting in limiting the functional divergence after duplications and polyploidization of *ORP* genes (Table S2). Only two orthologous gene pairs (GH_A02G0809 and Ga3G0877, GH_D02G0824 and Gorai.005G091400) have *Ka/Ks* ratio exceeding one, which implies these gene pairs underwent positive selection and had relatively rapid evolution rate.

### Gene structure and motif identification of *ORP* proteins

**Fig. 3:**
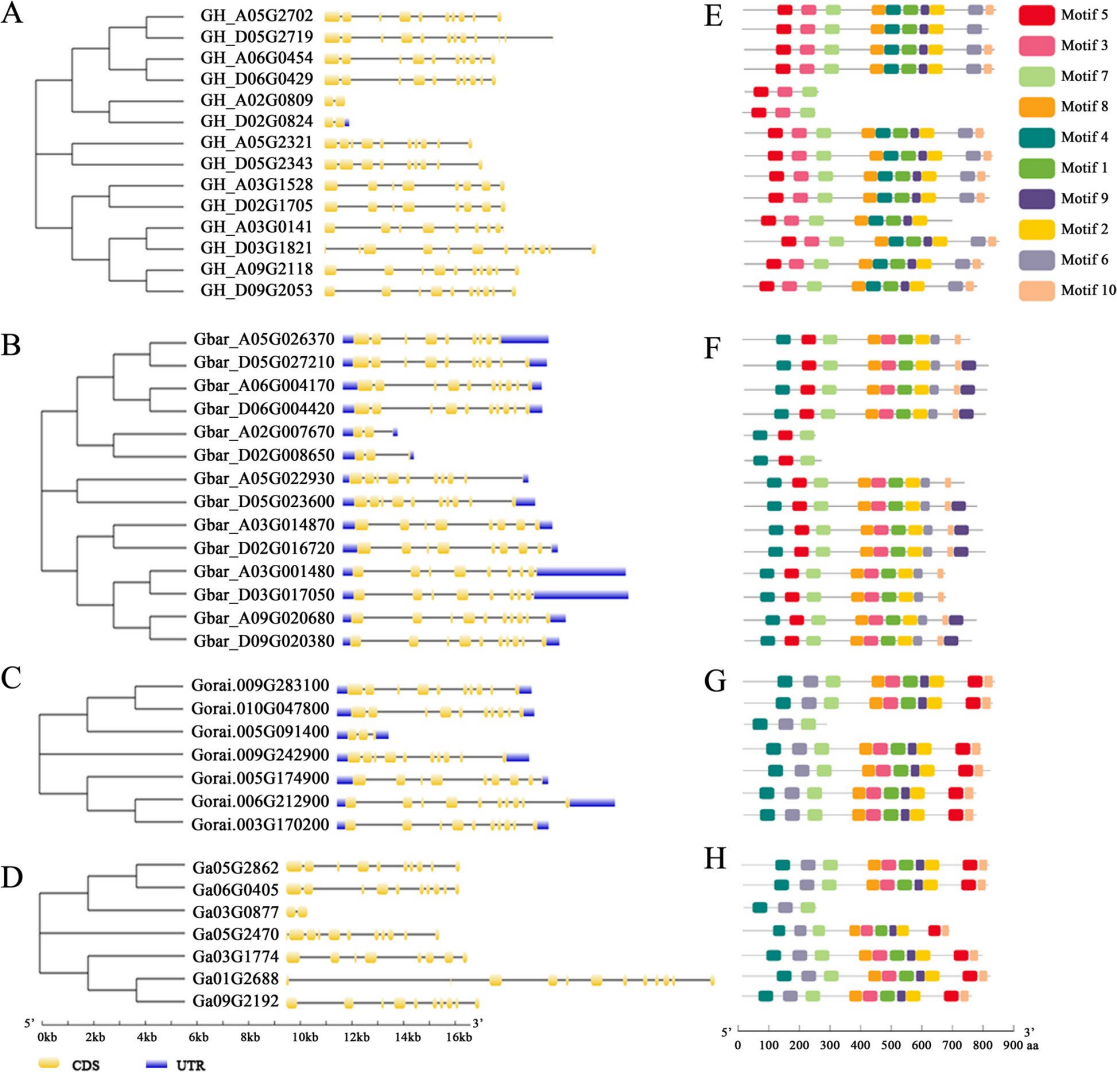
Gene structures and conserved motifs of all *ORP* genes in four cotton species. **A** and **E** *G. hirsutum*, **B** and **F** *G. barbadense*, **C** and **G** *G. arboreum*, **D** and **H** *G. raimondii*

The gene structure of *ORP* genes was analyzed according to the annotation files (Fig. 3A-D). Most *ORP* genes have 8–10 exons and only six genes in four cotton species have two or three exons. Genes classified into the same evolutionary branch have conserved gene structure patterns in terms of exon number and exon length. The MEME search identified ten conserved motifs in *ORP* genes, ranging from 300 to 2100 amino acids (Fig. 3E-H). The conserved motif numbers in different genes varied from 3 to 10. Motifs 2, 5, and 7 were conserved in all *ORP* genes in *G. hirsutum*. In *G. barbadense*, the conserved motifs were 4, 5, and 7, while in *G. arboreum* and *G. raimondii*, the conserved motifs were 4, 6, and 7.

### Identification and analysis of *cis*-acting elements

*Cis*-acting regulatory elements play a key role in molecular switches that control a dynamic gene activity network that initiates many biological processes, such as hormone responses, developmental processes, and abiotic stress responses [37]. MBS (drought inducibility), ABRE (abscisic acid-responsive), and TC-rich repeats (repeat cis-actin), which are involved in defense stress response and drought stress, could be found in all four cotton species (Figure S1). CAT-Box (meristematic cell expression), GARE-motif (Gibberellin responsive), and TGA-elements (auxin-responsive element), which are involved in the germination and regeneration stage, could also be found in all four cotton species.

### Expression profiles of *ORP* genes in *Gossypium hirsutum*

**Fig. 4:**
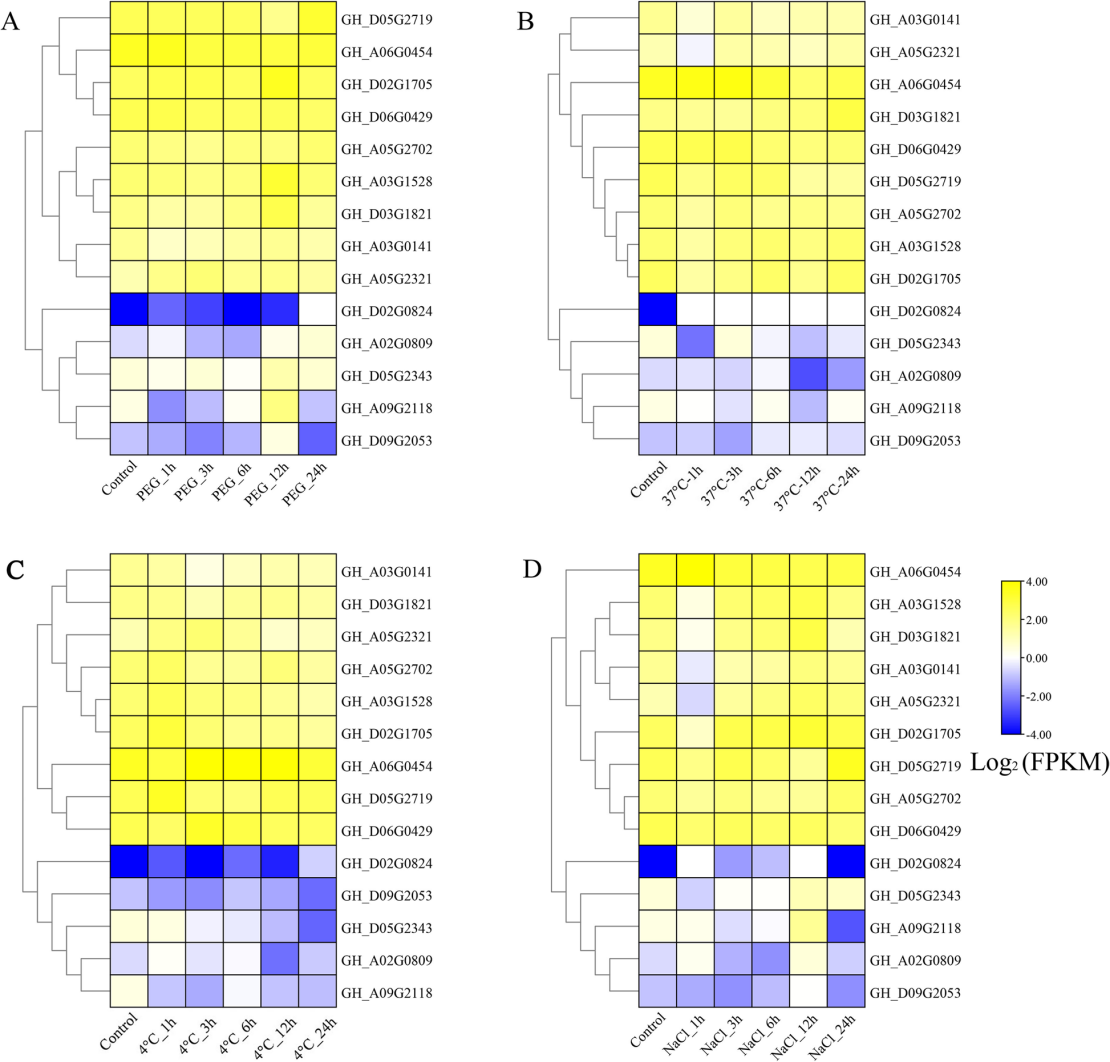
RNA-seq data analysis of *GhORP* genes under different treatments. **A** drought, **B** heat, **C** cold, **D** salt

The raw RNA-seq data of the 14 *ORP* genes in *G. hirsutum* were normalized to log_2_(FPKM), and the heatmap of the expression is presented as Fig. 4. Four genes, including *GH_A02G0809*, *GH_D02G0824*, *GH_A09G2118*, and *GH_D05G2343*, were up-regulated by the abiotic stress treatment. Especially, *GH_A02G0809* (*GhORP_A02*) expression was induced significantly by PEG treatment. We further performed experiments to characterize the function of *GhORP_A02* in drought stress.

### Virus-induced gene silencing of *GhORP_A02* in cotton show significant sensitivity to drought

**Fig. 5:**
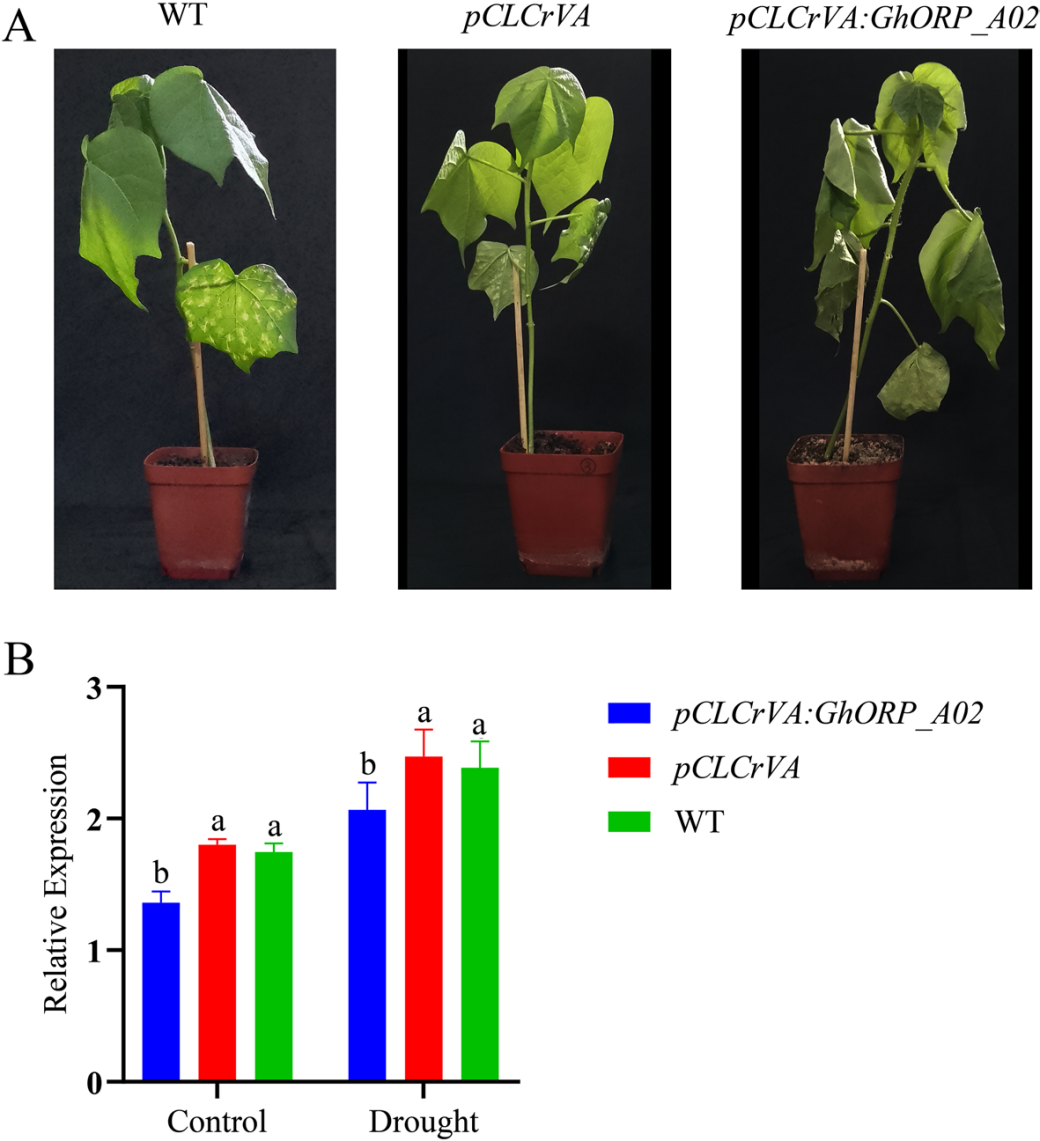
Virus-induced gene silencing of *GhORP_A02* in upland cotton. **A** Phenotypes of wild type, negative (*pCLCrVA*), and silenced plants (*pCLCrVA: GhORP_A02*) after drought treatment. **B** qRT-PCR analysis of wild type, silenced and control cotton plants after 10 days of drought treatment. Different letters indicated significant difference at *p* < 0.05

**Fig. 6:**
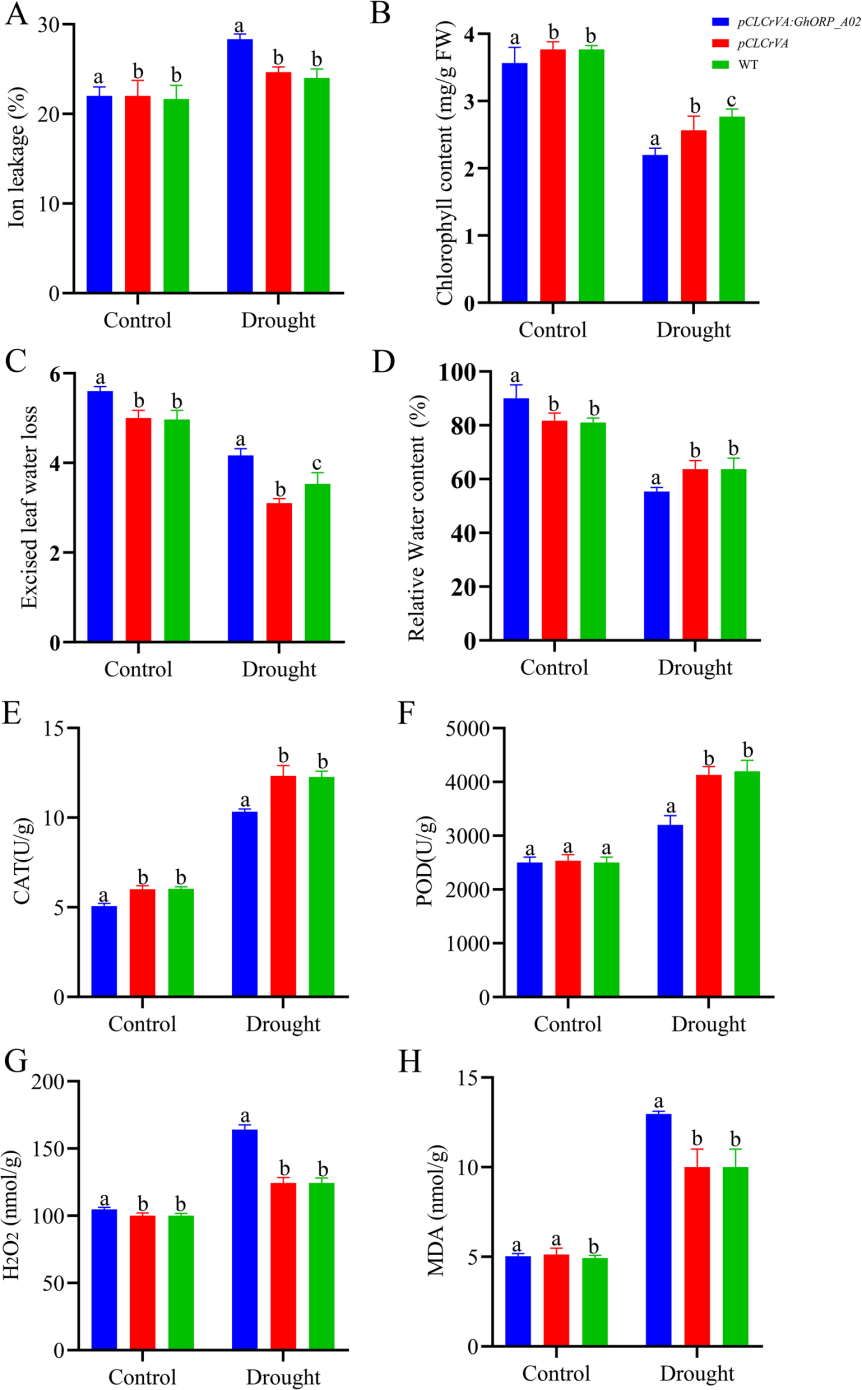
Physiological and enzyme activity analysis of *GhORP_A02* silenced plants. **A** Ion leakage, **B** Chlorophyll content, **C** Excised leaf water lost, **D** Relative leaf water content, **E** catalase, **F** POD, **G** H_2_O_2_, **H** MDA. Different letters indicated significant difference at *p* < 0.05

The method for gene silencing through virus-induced was used to analyze the role of the *GhORP_A02* in drought tolerance. *Gossypium hirsutum* acc. H177 was infected with three vectors, including *pCLCrVA: PDS* (positive control), *pCLCrVA* (negative control), and *pCLCrVA: GhORP_A02*. Ten days after infection, the indicator *pCLCrVA: PDS* showed albino color, the control plant showed a normal color without visible change, and the *pCLCrVA: GhORP_A02* plants showed complete shrinkage of the leaves, which indicates that VIGS was successful (Fig. 5A). qRT-PCR was used to analyze the expression level of *GhORP_A02* in silenced plants, and the result showed that the infected plant (*pCLCrVA: GhORP_A02*) showed a lower expression level than the control plant (Fig. 5B). The physiological analysis includes ion leakage, chlorophyll contents, excised leaf water loss, and relative leaf water content was done in silenced and controlled plants with and without drought treatment. The relative ion leakage level of the silenced plants increased by 20% compared to the control. The chlorophyll contents of the silenced plant were significantly lower in comparison to control plants. While in excised leaf water loss, the silenced plants lost more water than the control plant. The relative leaf water content of the silenced plant didn’t show a significant difference when compared to the control under drought conditions (Fig. 6A-D). Determination of antioxidant (CAT and POD) and oxidant (MDA and H_2_O_2_) enzyme concentration levels were analyzed in both control and silenced plants under drought conditions. There is a significant increase in the concentration of antioxidants and a decrease in the concentration of H_2_O_2_ in silenced plants compared with their respective control (Fig. 6E-H).

## Discussion

Drought is one of the most significant abiotic stresses, resulting in considerable yield losses in cotton [23, 40]. Plants have evolved self-defense systems to deal with abiotic stresses, which involves the transcription of stress-related genes [39]. Genetic enhancement of drought tolerance hinges on identifying genes related to drought tolerance [53]. In earlier research, drought-responsive genes were identified in many species like rice, peanut, soybean, wheat, maize and cotton [9, 21, 36, 44, 54]. Oxysterol-binding protein (ORP) and its homologs constitute a protein family in many eukaryotes, from yeast to humans, which are involved in cellular lipid metabolism, vesicle transport and signal transduction [51]. Recent studies have demonstrated that the *ORP* gene family was stress-responsive in various plants [31, 48, 43, 45]. Our current research has demonstrated the function of *GhORP_A02* in drought stress response. This study used the protein domain PFAM 15,413 to retrieve *ORP* genes in the four cotton species, and *G. hirsutum*, *G. barbadense*, *G. raimondii*, and *G. arboreum* encoding 14, 14, seven, and seven *ORP* genes, respectively. In previous research, 12 and six *ORP* genes were identified in *Arabidopsis* and rice, respectively [48]. Gene structure and phylogenetic tree analyses indicated that all *GhORP* genes, classified into one group, have a similar gene structure. The evolution analysis of the *ORP* gene in four cotton species shows no negative selection across all the species.

Conserved domains correspond conformational changes due to binding [28, 50]. Domain rearrangement and recombination, which typically occurs due to gene duplication and fission or fusion events, are used to develop new protein functions [38]. In this study, we identified two conserved motifs, motifs 5 and 7 in *G. hirsutum* and *G. barbadense*, while motif 7 was conserved in all four *Gossypium* species. Subcellular localization and the transcription of a gene under stress are powerful mechanisms to explain its biological function. *ORP* genes have been identified in *Arabidopsis*, soybean, rice, and Petunia and found to be located in plasm membrane, nucleus, and endoplasmic reticulum [31, 48, 43]. According to the subcellular localization prediction result, most *ORP* genes are located in the nucleus. Expression analysis of *GhORP* genes under different stress showed four genes were strongly induced by cold and drought stress from 1 to 24 h. A similar result was reported in soybean and *Arabidopsis*. For example, *GmOSBP* was induced by salt stress, and *AtORP4A* and *AtORP4B* were induced by drought stress [31, 48]. Expression analysis showed that four *GhORP* genes, especially *GhORP_A02*, were significantly up-regulated after drought stress, and we consider this gene as the candidate gene for drought stress response.

To investigate the function of *GhORP_A02*, we silenced this gene by VIGS. Resultantly, silenced plants were more sensitive to PEG treatment than control. Environmental challenges such as drought, salt and temperature cause a redox imbalance in plant cells, which rises the total rate of metabolism and finally up-regulates H_2_O_2_ production [17]. There is still no relative study about the mechanism of ORP proteins to cope with abiotic stress. How ORP proteins are involved in stress response is largely unknown. When plants are subjected to abiotic stress, membrane proteins degrade, and comparative conductivity and MDA are significantly elevated [12, 25]. In this study, electrolytes in silenced plants (*pCLCrVA: GhORP_A02*) increase significantly under drought stress compared to control plants. Both chlorophyll content and relative water contents decrease significantly in silenced plants, and this is in agreement with many previous research findings, which indicated that plants tend to close stomata to avoid water loss and decrease photosynthesis in drought conditions [22, 29, 46]. The reactive oxygen system produces substances such as POD and CAT, which are accompanied by an increase in reactive oxygen to limit and regulate the damage of reactive oxygen to plants, but also as a signal molecule to activate the plant body to respond to the external adverse environment [2, 3]. Our present work showed that Both CAT and POD decreased in the *pCLCrVA: GhORP_A02* plants when compared to control plants, and this signifies the signaling role of the *ORP* gene in enzymatic activity in cotton, which is consistent with previous research [33, 42].

## Conclusion

In this research, we carried out genome-wide identification, and a total of 42 *ORP* genes were distributed in *G. hirsutum*, *G. barbadense*, *G. arboreum*, and *G. raimondii*. All genes showed one-to-one homology relationships among *G. hirsutum*, *G. arboreum*, and *G. raimondii*. Gene structure and phylogenetic analysis indicated that *ORP* genes classified into one clade have similar structures. Analysis of *ORP* genes in four *Gossypium* species revealed that most proteins are localized in the nucleus. The *Ka/Ks* ratio between orthologous gene pairs revealed that *ORP* genes had undergone purifying and positive selection during evolution. We also identified ABA, GA, auxin and drought stress response elements in promoter regions. Further expression analysis using transcriptome data indicated that four *GhORP* genes were highly expressed after abiotic stress treatment. Characterization of *GhORP_A02* through virus-induced gene silencing found that *GhORP_A02* participated in drought stress by inducing various physiological and biochemical changes. Our study provided a useful reference for further functional investigation of *GhORP* genes.

## Supplementary Information

**Additional file 1: Supplementary Table 1.** Primers used in this study.

**Additional file 2: Supplementary Table 2.** Ka/Ks analysis of duplicated ORP gene pairs of G. hirsutumm, G.raimondii and G. arboretum.

**Additional file 3: Figure S1.** *Cis*-acting elements identified in promoter regions of ORP genes.

## Data Availability

Genome sequences of *G. hirsutum* acc. TM-1 (ZJU_V2.1), *G. barbadense* acc.3–79 (HAU_V2.0), *G. arboreum* (CRI_V3.0) and *G. raimondii* (JGI_V2.1) are available in the CottonFGD website (https://cottonfgd.net/about/download.html). Transcriptome data of TM-1 was downloaded from NCBI Sequence Read Archive under the accession number SRA180756.

## Abbreviations

ORP: Oxysterol-binding protein-related proteins
VIGS: Virus-induced gene silencing
ROS: Reactive oxygen species
*G. arboreum*: *Gossypium arboreum*
*G. hirsutum*: *Gossypium hirsutum*
*G. barbadense*: *Gossypium barbasense*
*G. raimondii*: *Gossypium raimondii*
MW: Molecular weight
NJ: Neighbor-joining
ML: Maximum likelihood
Ka: Non-synonymous
Ks: Synonymous
FPKM: Fragments per kilobase of exon per million fragments mapped
qRT-PCR: Quantitative real-time PCR
ABA: Abscisic acid
JA: Jasmonic acid
POD: Peroxidas
MDA: Malondialdehyde
CAT: Catalase
