# Supplementary material for: Silencing of GhORP_A02 enhances drought tolerance in Gossypium hirsutum
Source: BMC Genomics. 2023 Jan 9;24:7. doi: 10.1186/s12864-022-09099-y (PMC9830788; doi:10.1186/s12864-022-09099-y)
Supplement: Supplementary file 1 — Additional file 1: Supplementary Table 1. Primers used in this study. [file 12864_2022_9099_MOESM1_ESM.docx]

Supplementary table 1: Primers used in this study

| Primer ID | Sequence(5'-3') | Restriction sites | Purpose |
| --- | --- | --- | --- |
| qPCR_ GH_A02G0809F | CGTTGCAGAAGCAGCTCGTA |  | qRT-PCR |
| qPCR_ GH_A02G0809R | ACCGATAATTTCCTCTCATGTTCA |  | qRT-PCR |
| Histone3F | TCAAGACTGATTTGCGTTTCCA |  | Internal control |
| Histone3R | GCGCAAAGGTTGGTGTCTTC |  | Internal control |
| VIGS_ GH_A02G0809F | ATGCCTGCAGACTAGTGCCCTCTTGCTCCAAAGCTA | *Spe* I | Constructing vector for VIGS |
| VIGS_ GH_A02G0809R | AGACCTAGGGGCGCGCCTCTCCCGTGCTTTGTGGTTT | *Asc* I | Constructing vector for VIGS |
