# Supplementary material for: Silencing of GhORP_A02 enhances drought tolerance in Gossypium hirsutum
Source: BMC Genomics. 2023 Jan 9;24:7. doi: 10.1186/s12864-022-09099-y (PMC9830788; doi:10.1186/s12864-022-09099-y)
Supplement: Supplementary file 2 — Additional file 2: Supplementary Table 2. Ka/Ks analysis of duplicated ORP gene pairs of G. hirsutumm, G.raimondii and G. arboretum. [file 12864_2022_9099_MOESM2_ESM.doc]

Supplementary Table 2: Ka/Ks analysis of duplicated *ORP* gene pairs of *G. hirsutumm*, *G.raimondii* and *G. arboretum*.

| Seq_1 | Seq_2 | Ka | Ks | Ka/Ks |
| --- | --- | --- | --- | --- |
| GH_A02G0809 | Ga03G0877 | 0.0035 | 0 | ∞ |
| GH_A03G0141 | Ga01G2688 | 0.0067 | 0.0098 | 0.6892 |
| GH_A03G1528 | Ga03G1774 | 0.0038 | 0.0074 | 0.5064 |
| GH_A05G2321 | Ga05G2470 | 0.0022 | 0.0097 | 0.2247 |
| GH_A05G2702 | Ga05G2862 | 0.0031 | 0.0109 | 0.2893 |
| GH_A06G0454 | Ga06G0405 | 0.0005 | 0.0056 | 0.0947 |
| GH_A09G2118 | Ga09G2192 | 0.0017 | 0.0039 | 0.4396 |
| GH_D02G0824 | Gorai.005G091400 | 0.0090 | 0.0066 | 1.3653 |
| GH_D02G1705 | Gorai.005G174900 | 0.0011 | 0.0037 | 0.2899 |
| GH_D03G1821 | Gorai.003G170200 | 0.0023 | 0.0156 | 0.1443 |
| GH_D05G2343 | Gorai.009G242900 | 0.0005 | 0.0039 | 0.1402 |
| GH_D05G2719 | Gorai.009G283100 | 0.0188 | 0.0286 | 0.6591 |
| GH_D06G0429 | Gorai.010G047800 | 0.0021 | 0.0149 | 0.1415 |
| GH_D09G2053 | Gorai.006G212900 | 0.0045 | 0.0078 | 0.5844 |
| Ga01G2688 | Gorai.003G170200 | 0.0051 | 0.0316 | 0.1609 |
| Ga03G0877 | Gorai.005G091400 | 0.0170 | 0.0230 | 0.7402 |
| Ga03G1774 | Gorai.005G174900 | 0.0059 | 0.0302 | 0.1963 |
| Ga05G2470 | Gorai.009G242900 | 0.0071 | 0.0358 | 0.1994 |
| Ga05G2862 | Gorai.009G283100 | 0.0084 | 0.0293 | 0.2866 |
| Ga06G0405 | Gorai.010G047800 | 0.0042 | 0.0320 | 0.1320 |
| Ga09G2192 | Gorai.006G212900 | 0.0109 | 0.0332 | 0.3288 |
