# Supplementary material for: Silencing of GhORP_A02 enhances drought tolerance in Gossypium hirsutum
Source: BMC Genomics. 2023 Jan 9;24:7. doi: 10.1186/s12864-022-09099-y (PMC9830788; doi:10.1186/s12864-022-09099-y)
Supplement: Supplementary file 3 — Additional file 3: Figure S1. Cis-acting elements identified in promoter regions of ORP genes. [file 12864_2022_9099_MOESM3_ESM.docx]

**
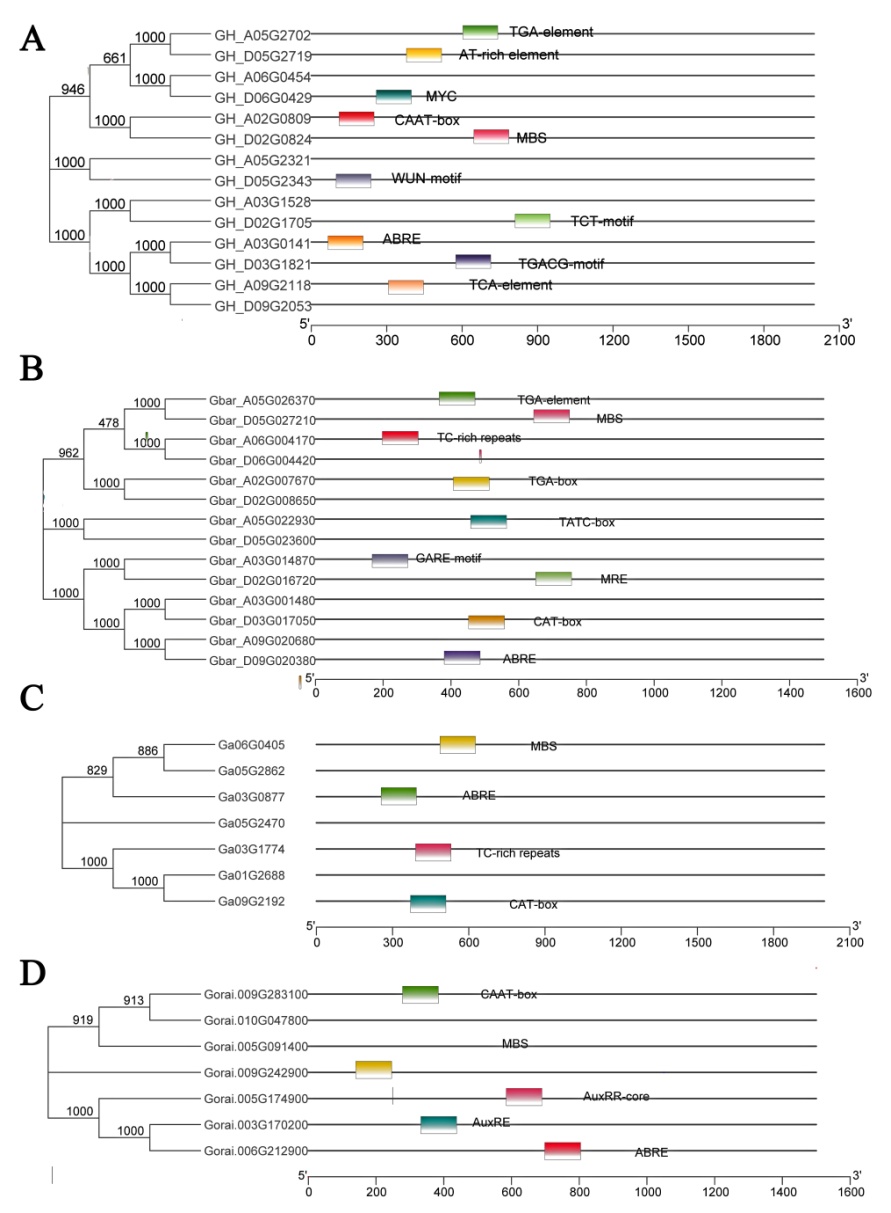
Figure S1** *Cis*-acting elements identified in promoter regions of *ORP* genes.

A: *G. hirsutum*, B: *G. arboretum*, C*: G. barbadense*, D: *G. raimondii*.
